# Enhancing the Resolution Utilization for DIC Measurement of Slender Components Using Shear Imaging

**DOI:** 10.3390/s25237346

**Published:** 2025-12-02

**Authors:** Yinhang Ma, Kangjiang Lv, Zhuoxuan Song, Dong Jiang

**Affiliations:** School of Mechanical and Electronic Engineering, Nanjing Forestry University, Nanjing 210037, China; lvkangjiang3610@njfu.edu.cn (K.L.); zhuoxuansong@njfu.edu.cn (Z.S.); jiangdong@njfu.edu.cn (D.J.)

**Keywords:** digital image correlation, Michelson shear device, slender object, resolution utilization

## Abstract

Digital image correlation (DIC) is widely used for full-field deformation measurement, yet its spatial resolution is often underutilized when measuring slender components due to their high aspect ratio. To address this limitation, a novel virtual multi-camera DIC method that integrates a conventional dual-camera setup with two Michelson shear devices (MSDs) was proposed. Each MSD splits the image of the slender component along its longitudinal direction, projecting two segments side-by-side onto the same camera sensor. This configuration effectively enhances the resolution utilization of each camera, enabling high-resolution measurement of the entire slender surface without requiring additional cameras. The system is calibrated to establish extrinsic parameters between DIC subsystems, allowing stitching of 3D data from different regions. Experimental validation through translation and bending tests demonstrates that the proposed method achieves accurate full-field morphology and deformation measurements, with sub-pixel level agreement in overlapping regions. This approach offers a practical and cost-effective solution for enhancing DIC performance in constrained measurement environments.

## 1. Introduction

The stereo vision-based digital image correlation (stereo-DIC) method is a powerful and practical non-contact optical measurement technique for full-field surface topography and deformation [1]. Due to its advantages of simplicity in equipment setup, ease of implementation, wide applicability, and high measurement accuracy, stereo-DIC has found extensive applications in fields of civil engineering [2], transportation [3], aerospace [4], etc. In practical engineering measurements, it is common to encounter a variety of slender-shaped objects, like beams and columns. When these are measured for deformation using a dual-camera system, achieving complete coverage of the entire slender surface with the camera’s field of view necessitates a large loss of spatial resolution along the smaller dimensions [5]. Low image resolution means low measurement sensitivity for DIC method; therefore, improving the utilization of resolution becomes critically important when measuring slender objects.

To enhance the system resolution for measuring the surface topography and deformation field of slender objects, researchers have developed a multi-camera DIC (MC-DIC) system, which divides the object into several regions, and each region is measured by a dedicated DIC subsystem [6,7,8,9,10,11,12]. These measurement data from different subsystems are then transformed into a unified coordinate system by the calibration method [13,14] to acquire comprehensive data of the slender object. Typically, the slender objects studied are of meter or decimeter scale, found in large engineering structures where the measurement environment has ample space allowing the multiple cameras to be optimally positioned. Conversely, the minor dimensions of slender objects referred to as slender components in mechanical systems typically range from centimeters to decimeters, necessitating that test cameras be positioned close to the object to resolve details. This proximity results in a testing environment that lacks sufficient space to accommodate multiple cameras. An effective strategy to address this challenge is to transform a single camera into two virtual cameras, which is achieved by employing optical devices to generate two distinct viewpoints.

Several methods have been developed to realize this concept. One approach involves the use of a bi-prism mounted in front of the camera lens. The prism refracts incoming light, splitting the scene into two separate images projected onto the left and right halves of the sensor, simulating a stereo pair [15]. This setup is exceptionally compact, ideal for confined spaces, but introduces significant image distortion that demands complex, model-free correction techniques for accurate results. A more robust and widely applicable technique employs a four-mirror adapter to direct two separate optical paths onto the camera sensor [16]. The planar mirrors induce negligible distortion, making the accuracy of this system comparable to a conventional two-camera stereo-DIC setup. The full-frame color camera-based method uses a color-separation apparatus (e.g., a beam splitter with filters and mirrors) to encode the two viewpoints into different color channels (e.g., red and blue) [17]. These views are captured over the entire sensor area simultaneously, thus fully preserving its spatial resolution. Both the four-mirror adapter and the color camera solutions have inherent limitations. Their setups involve numerous components and exhibit multiple degrees of freedom, making them difficult to adjust. Furthermore, their fixed optical configurations make them unsuitable for segmenting slender structures imaged on the sensor. Therefore, effectively increasing the resolution utilization based on the traditional stereo-DIC system is of great significance for the measurement of slender components.

This study proposes a novel method for nearly doubling the camera resolution utilization of DIC system measuring the slender components by using Michelson shearing devices. The Michelson shear device consists of a beam splitting prism and two mirrors. Widely used in speckle shear interferometry, the Michelson shearing device enables the formation of dual images of an object on one camera target [18]. By employing a single Michelson shearing device and camera, the structure’s image on the focal plane can be divided into two sections along its length. By adjusting the mirrors, these sections are positioned side by side along the width, allowing for image segmentation and stitching to produce a complete image of the elongated structure. Employing two sets of Michelson shearing device and two cameras can thus create a dual DIC system. This not only reduces the requirements for space and the number of cameras in the multi-camera system, but also significantly improves the utilization rate of the camera resolution in the dual-camera stereo-DIC system.

The remainder of this paper is organized as follows. Section 2 elaborates on the methodology of the proposed multi-camera DIC system, including the optical setup based on Michelson shear devices, the principle of virtual camera formation, system calibration, and the data stitching strategy. Section 3 presents experimental validation, including a translational test on a ceramic plate and a bending test on a cantilever beam, to verify the accuracy and practicality of the method. Finally, Section 4 summarizes the main conclusions and contributions of this work.

## 2. Method

In mechanical systems, the aspect ratio of slender components typically exceeds 5, while the aspect ratio of a camera’s image sensor is usually between 1~2. When using a single-setup DIC system for measurement of a slender component, a large part of pixels must be wasted to ensure the camera’s field of view covers the entire length of the component. Theoretically, using two DIC systems to measure each half of the slender structure can double the utilization rate of the camera’s resolution. However, this approach introduces two practical issues: firstly, a substantial increase in hardware costs, and secondly, the limited space in front of the slender components does not allow for additional cameras. Therefore, enhancing the resolution utilization on the existing single-setup DIC system is essential.

**Figure 1:**
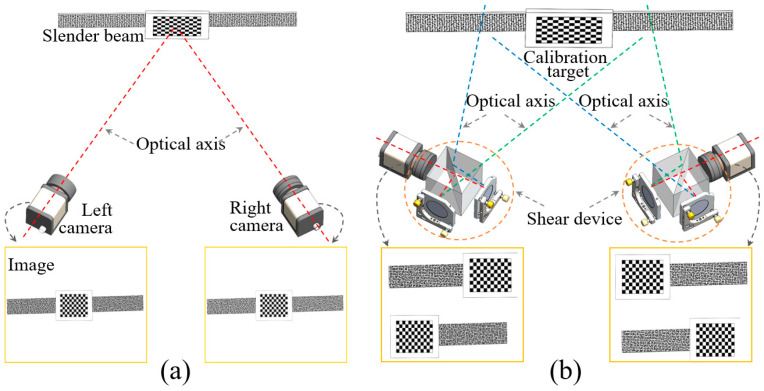
Digital image correlation (DIC) system: (**a**) a traditional dual-camera DIC system; (**b**) a dual DIC system equipped with two shear devices.

Figure 1 serves to visually contrast the fundamental differences in both hardware configuration and imaging principles between a conventional stereo-DIC system and our proposed Michelson shear device (MSD)-based system. Figure 1a depicts the standard setup, which comprises two physical cameras that directly image the specimen from different viewpoints. Each camera sensor captures a single, continuous image of the entire object, but this setup results in poor resolution utilization when measuring slender components, as a significant portion of the sensor area is occupied by the background to accommodate the full length of the object.

In contrast, our novel system, illustrated in Figure 1b, integrates an MSD into the optical path in front of each physical camera. The core innovation lies in its imaging principle: each MSD splits the light from the object, shearing and directing rays from different parts of the component to different regions of the camera sensor. This process effectively transforms a single physical camera into two “virtual cameras”, creating two distinct, adjacent fields of view on the same sensor. This configuration allows for the entire length of slender components to be captured with nearly double the resolution utilization, compared to the conventional system. Furthermore, by using another set of MSDs and cameras from different angles, the full view of the slender structure can be captured from alternative perspectives.

A typical MSD consists of two mirrors and a beam-splitting prism, which is used in speckle interferometry to produce two misaligned images on the camera’s image plane. These mirrors are mounted on a two-dimensional adjustment frame, allowing for the repositioning of the misaligned images. The system effectively records the entire length of a slender structure with one camera, nearly doubling the resolution utilization.

**Figure 2:**
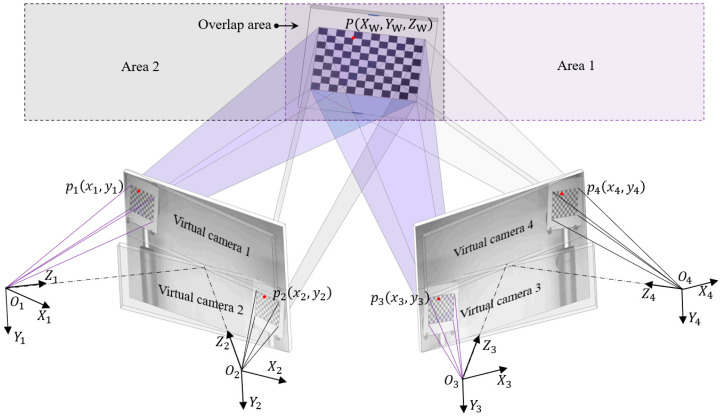
Schematic of the multi-camera DIC method.

Figure 2 presents the schematic diagram of the MC-DIC system. Thanks to the MSDs, the image plane of the real camera is divided into upper and lower sections. Consequently, two cameras are transformed into four virtual cameras, with the optical center coordinate (OCC) systems designated as $O_{1}{-X}_{1}Y_{1}Z_{1}$, $O_{2}{-X}_{2}Y_{2}Z_{2}$, $O_{3}{-X}_{3}Y_{3}Z_{3}$, and $O_{4}-X_{4}Y_{4}Z_{4}$, respectively. Cameras 1 and 3 together form DIC subsystem 1 (DIC1), responsible for measuring Area 1, while cameras 2 and 4 comprise DIC subsystem 2 (DIC2), tasked with measuring Area 2. Areas 1 and 2 share an overlapping region that can accommodate a checkerboard calibration target. When the calibration target is placed in the overlapping region, all four virtual cameras can simultaneously capture the board, allowing the target to serve as a medium for establishing the relative extrinsic parameters (i.e., translation and rotation matrices) between the two DIC subsystems. Based on these relative extrinsic parameters, the 3D data measured by the two DIC subsystems can be integrated within the same coordinate system, providing complete data for measuring slender regions.

**Figure 3:**
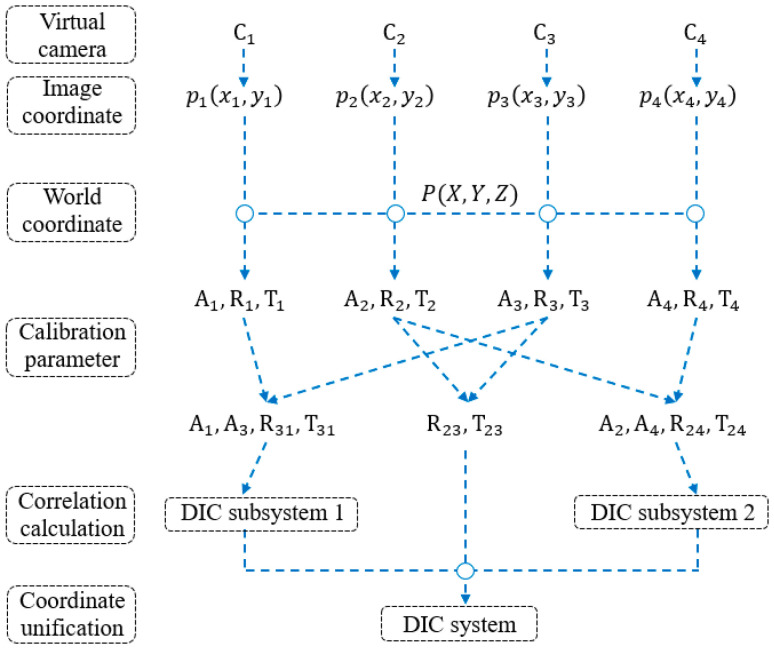
Calculation process of multi-camera DIC system.

The computational process of the proposed multi-camera DIC measurement method is illustrated in Figure 3 and mainly consists of the following steps:

(1) Calibration of the intrinsic and extrinsic parameters for the four virtual cameras.

As depicted in Figure 2, virtual cameras 1 and 3 form a stereo vision model, and virtual cameras 2 and 4 form another stereo vision model. The OCC systems for virtual cameras are $O_{i}{-X}_{i}Y_{i}Z_{i}$, the subscript i is the virtual camera number, and i = 1,2,3,4. The world coordinate (WC) system is represented as $O_{W}{-X}_{W}Y_{W}Z_{W}$. For the DIC subsystem 1 composed by virtual camera 1 and camera 3, a point marked by $P(X_{W},Y_{W},Z_{W})$ in the object space is represented in the optical center coordinate systems of camera 1 as $P_{1}(X_{1},Y_{1},Z_{1})$ and camera 3 as $P_{3}(X_{3},Y_{3},Z_{3})$. The corresponding image coordinates in camera 1 and camera 3 are $p_{1}(x_{1},y_{1})$ and $p_{3}(x_{3},y_{3})$, respectively. Through coordinate transformation, the image coordinates and world coordinates of a point are related as follows:

(1) $$Z_{i}\left[\begin{array}{c} x_{i}\\ y_{i}\\ 1 \end{array}\right]=A_{i}\left[R_{i}T_{i}\right]\left[\begin{array}{c} X_{W}\\ Y_{W}\\ Z_{W}\\ 1 \end{array}\right]$$

where $A_{i}$ represents the intrinsic parameters of camera i. $R_{i}$ and $T_{i}$ are the extrinsic parameters of camera i, which are used to describe the transformation relationship between the OCC system and the WC system. The intrinsic and extrinsic parameters of the camera are generally obtained through Zhang’s calibration method [14]. The relationship between the OCC systems of camera 1 and camera 3 consisting of DIC subsystem 1 can be expressed as follows:

(2) $$\begin{array}{c} \left[\begin{array}{c} X_{3}\\ Y_{3}\\ Z_{3} \end{array}\right]=\left[R_{3}R_{1}^{T}\right]\left[\begin{array}{c} X_{1}\\ Y_{1}\\ Z_{1} \end{array}\right]+T_{3}-R_{3}R_{1}^{T}T_{1}\\ P_{3}=R_{31}P_{1}+T_{31} \end{array}$$

where $R_{31}$ and $T_{31}$ describe the relationship between the OCC systems of cameras 1 and 3. $R_{13}$ represents the rotational matrix, and $T_{13}$ is the translation matrix. These extrinsic parameters also can be determined through the calibration method. In the same way, the corresponding intrinsic and extrinsic parameters for DIC subsystem 2, which consists of cameras 2 and 4, are derived. Subsystem 1 designates camera 2 as its respective master camera, and Subsystem 2 designates camera 3 as its respective master camera. Therefore, the relative extrinsic parameters between subsystem 1 and subsystem 2, which represent the transformation relationship between the OCC systems of camera 3 and camera 2, are also obtained.

(2) Build the DIC subsystems 1 and 2 and measure the 3D data of slender component.

Following Step 1, which involves calibrating the intrinsic and extrinsic parameters of the cameras, DIC subsystem 1 and subsystem 2 are set up. Taking subsystem 1 as an example, the 3D spatial coordinates of a point are then reconstructed from its 2D image coordinates captured by the two cameras, based on their intrinsic and extrinsic parameters. During deformation measurement, a reference image sub-area is selected from the pre-deformation reference image. Using search methods, template matching is performed on the target image post-deformation, following a specific correlation function. This process seeks the target image sub-area with the highest correlation to the reference image sub-area, thereby determining the displacements of the measurement points in various directions.

(3) Unify the data of the two subsystems in the same coordinate system.

Subsystem 1 and subsystem 2 measure different regions of slender structures, each operating under different coordinate systems. Through the calibration in Step 1, the transformation relationship between the OCCs of cameras 2 and 3 is determined. Utilizing this relationship allows us to unify the data from both subsystems within the same coordinate system.

## 3. Experiment

This section describes the experimental validation of the proposed multi-camera DIC system for morphological and deformation measurements through in-lab experiments: a translational experiment on a ceramic plate and a bending test on a cantilever beam, conducted on an isolation table.

(1) Ceramic Plate Translational Experiment

**Figure 4:**
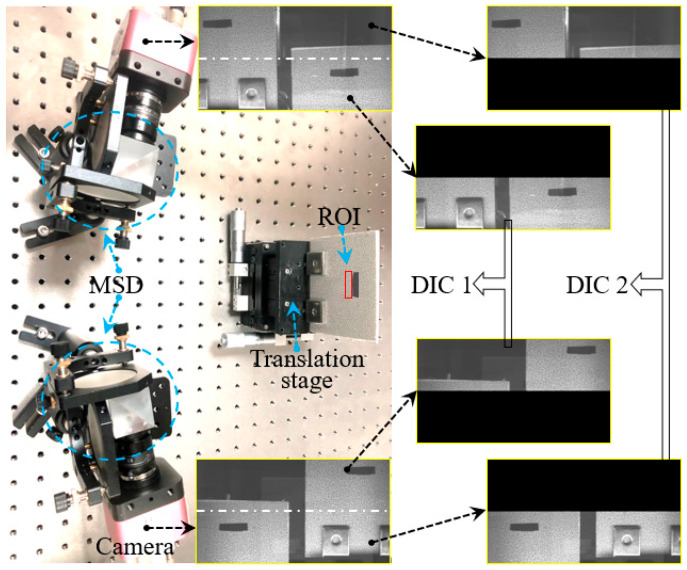
Experimental system based on MSD.

A virtual MC-DIC system was set up, as shown in Figure 4. The system comprises two industrial cameras, a computer, and two MSDs. The cameras are the MV1-D2040×1088-240-CL-8 models, providing a resolution of 2040 × 1088 pixels (Photonfocus AG, Lachen, Switzerland). Capable of capturing up to 85 frames per second at full resolution, these cameras are paired with 30 mm focal length lenses. The cameras are interfaced with an image acquisition card via the Camera Link connection. The server is equipped with an Intel(R) Xeon(R) Gold 5122 processor running at 3.6 GHz, 64 GB of memory, and a 2 TB solid-state drive (KETIANJIAN Tech., Ltd., Changsha, China). A set of MSD consists of two reflecting mirrors and a beam-splitting prism. The surface flatness of these lenses is better than one quarter of a wavelength (633 nm). Calibration of the system’s intrinsic and extrinsic parameters was carried out using a 12 × 9^−3^ mm calibration plate. The reprojection error of subsystem 1 is 0.07 pixels, and the reprojection error of subsystem 2 is 0.06 pixels, both of which are below the commonly accepted precision threshold of 0.1 pixels in the DIC field [19]. This indicates that the systematic distortion introduced by MSD has been effectively compensated by the calibration model. The relative extrinsic parameters of subsystem 1 and subsystem 2 are as follows:

$$R_{23}=\left[\begin{array}{c} 0.9453\;0.0375\;0.3240\\ -0.0115\;0.9966-0.0817\\ -0.3260\;0.0735\;0.9425 \end{array}\right],\;T_{23}=\left[\begin{array}{c} -200.348\\ 6.840\\ 21.096 \end{array}\right]$$

**Figure 5:**
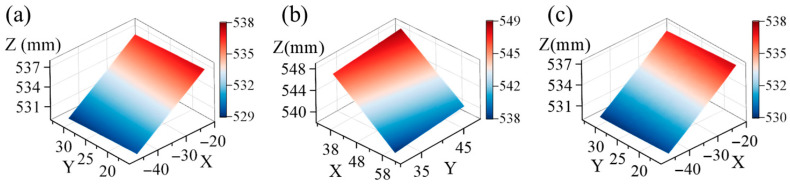
The morphology of the ceramic plate obtained from (**a**) subsystem 1, (**b**) subsystem 2, and (**c**) subsystem 2 after transformation.

The ceramic plate was coated with water-transfer speckles, with an average speckle diameter of about 3–5 pixels. It was mounted on a high-precision three-axis translation stage (precision of 5 μm) and positioned within the common field of view of four virtual cameras. The overlap area was approximately one-fifth of the total length of the image, and a region of 192 × 308 pixels was selected as the region of interest (ROI). Initially, the morphology of the ceramic plate was measured, as depicted in Figure 5. Figure 5a is obtained by subsystem 1, Figure 5b is obtained by subsystem 2, and Figure 5c is the transformed data of subsystem 2 to the OCC of subsystem 1.

Then, error statistics were compiled for both datasets within the same coordinate system. The three directional errors were calculated as

(3) $$e=\left[\begin{array}{c} e_{x}\\ e_{y}\\ e_{z} \end{array}\right]=\left[\begin{array}{c} x_{2}-x_{1}\\ y_{2}-y_{1}\\ z_{2}-z_{1} \end{array}\right]$$

**Figure 6:**
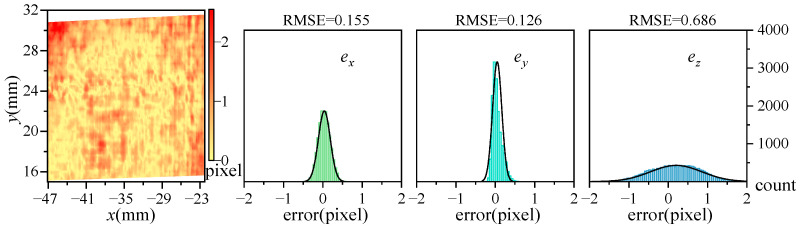
The error of subsystem 2 with respect to subsystem 1.

Figure 6 displays the errors outcomes when comparing three-dimensional data measured by Subsystem 2 against data from Subsystem 1: left, absolute error distribution; right, three directional errors statistics. The error in the y-direction is notably minimal, and the RMSE is 0.126 pixels. Errors in the x-direction are also small, and the RMSE is 0.155 pixels. The z-direction shows higher relative errors with an RMSE of 0.686 pixels, yet over 85% of data points maintain errors less than 1 pixel. These results affirm a strong agreement in the data from overlapping regions measured by the two subsystems.

**Figure 7:**
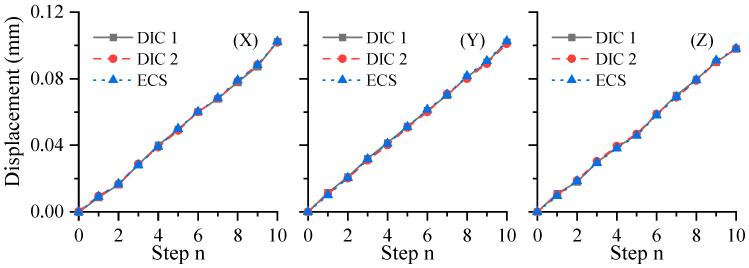
Displacement comparison.

During the translational experiments, a translation stage was employed to displace the ceramic plate along a predefined linear path. Throughout the motion, speckle images were simultaneously captured by both cameras to measure the pure translational displacement of the plate. For validation purposes, an eddy current sensor (ECS) with a sensitivity of 2 μm was used as a reference to monitor the displacement. Manual adjustments were made to the differential micrometer head on the translation stage, advancing the plate in ten sequential steps of 10 μm each. As illustrated in Figure 7, a comparative analysis reveals a high level of agreement between the displacement values obtained from the multi-camera DIC system and those recorded by the ECS, which demonstrate that the proposed method achieves a high precision.

In order to verify the impact of improved resolution on accuracy, we compared the experimental results with those obtained from the DIC system without the MSD. In our previous study [20], we used a DIC system without the MSD, which had the same components, including the camera, lens, translation stage, and test ceramic plate, as the system in this paper. Additionally, the translation process was the same, so we used the test data from that study for comparison. We analyzed the RMSE between the measurements from the two systems and the ECS measurements as shown in Table 1. The results show that the system proposed in this paper exhibits superior measurement accuracy in both the x- and z-directions compared to the DIC system without the MSD. In the y-direction, the measurement accuracies of both systems are comparable.

**Figure 8:**
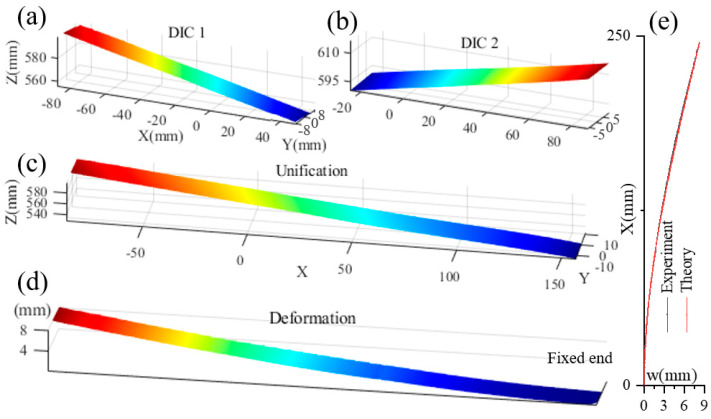
Morphology and deformation of a cantilever beam: (**a**) left segment morphology before deformed of cantilevered beam, measured by the DIC system 1; (**b**) right segment morphology before deformation of cantilever beam, measured by the DIC system 2; (**c**) the full morphology of beam before deformation; (**d**) the complete morphology of beam after deformation; (**e**) the deformation comparison of the complete beam between experiment and theory.

(2) Cantilever Beam Bending Experiment:

To further evaluate the practical performance of the proposed method in deformation measurement, a bending experiment was performed on a cantilever beam mounted on an isolation table to minimize external vibrations. The beam had a length of 240 mm, a width of 20 mm, and a thickness of 2 mm. Its surface was coated with a water-transfer speckle pattern to facilitate deformation analysis, and it was carefully positioned within the overlapping field of view of the multi-camera system. Subsystem 1 was assigned to capture the right side of the beam, while subsystem 2 monitored the left side, with a common overlapping region ensuring full-field data integration. A set of reference images was first acquired under unloaded conditions. Subsequently, a concentrated static load was applied at the free end of the beam, and deformed images were recorded. Following the computational procedures outlined earlier, the local deformation on the right side was determined using subsystem 1 data, and the left-side deformation was derived from subsystem 2. Both datasets were then transformed and merged into a unified global coordinate system. The resulting full-field deformation distribution of the cantilever beam is presented in Figure 8. Comparison of the experimental and theoretical results clearly demonstrates that the multi-camera DIC system is capable of accurately reconstructing the complete 3D morphology and capturing full-field deformation with high resolution utilization rate.

## 4. Conclusions

To overcome the limitation of low-resolution utilization in conventional dual-camera stereo-DIC systems when measuring mechanical components with large aspect ratios, this study proposes a novel virtual multi-camera stereo-DIC method by integrating a single-set DIC system with dual MSDs. Based on the conventional single-set DIC framework, the MSD-based image-splitting principle divides the slender component into two segments, which are simultaneously projected onto a single camera sensor. This configuration nearly doubles the resolution utilization of the camera. The feasibility and accuracy of the proposed method in capturing both 3D morphology and deformation were experimentally verified through translational tests on a ceramic plate and bending experiments on a cantilever beam. The results confirm its strong potential for high-resolution full-field deformation measurement of slender structures.

## Figures and Tables

**Table 1:** RMSE of displacement measured by the proposed system and traditional DIC system.

RMSE (µm)		X-Direction	Y-Direction	Z-Direction
Proposed system	DIC1	0.566	0.656	0.697
	DIC2	0.551	1.07	0.761
Traditional DIC [20]		0.95	0.94	1.48

## Data Availability

The data that support the findings of this study are available upon reasonable request from the authors.
